# Understanding the Properties of Starch in Potatoes (*Solanum tuberosum* var. Agria) after Being Treated with Pulsed Electric Field Processing

**DOI:** 10.3390/foods8050159

**Published:** 2019-05-10

**Authors:** Setya B.M. Abduh, Sze Ying Leong, Dominic Agyei, Indrawati Oey

**Affiliations:** 1 Department of Food Science, University of Otago, Dunedin 9016, New Zealand; setya.abduh@postgrad.student.ac.nz (S.B.M.A.); sze.leong@otago.ac.nz (S.Y.L.); dominic.agyei@otago.ac.nz (D.A.); 2 Department of Food Technology, Diponegoro University, Semarang 50275, Indonesia; 3 Riddet Institute, Palmerston North 4442, New Zealand

**Keywords:** potato starch, pulsed electric fields, birefringence, thermal properties, enzyme susceptibility

## Abstract

The purpose of this study was to investigate the properties of starch in potatoes (*Solanum tuberosum* cv. Agria) after being treated with pulsed electric fields (PEF). Potatoes were treated at 50 and 150 kJ/kg specific energies with various electric field strengths of 0, 0.5, 0.7, 0.9 and 1.1 kV/cm. Distilled water was used as the processing medium. Starches were isolated from potato tissue and from the PEF processing medium. To assess the starch properties, various methods were used, i.e., the birefringence capability using a polarised light microscopy, gelatinisation behaviour using hot-stage light microscopy and differential scanning calorimetry (DSC), thermal stability using thermogravimetry (TGA), enzyme susceptibility towards α-amylase and the extent of starch hydrolysis under in vitro simulated human digestion conditions. The findings showed that PEF did not change the properties of starch inside the potatoes, but it narrowed the temperature range of gelatinisation and reduced the digestibility of starch collected in the processing medium. Therefore, this study confirms that, when used as a processing aid for potato, PEF does not result in detrimental effects on the properties of potato starch.

## 1. Introduction

Pulsed electric field (PEF) processing has been reported to have a capability in modifying the microstructure of solid plant foods [1]. This leads to the reduction of the cutting force for potato tuber [2] and sweet potato [3] and the oil uptake of these commodities during frying. Another study by [4] has also found that PEF processing in combination with calcium chloride and trehalose solutions could retain the textural properties of frozen potatoes. Due to these benefits to improve product quality, this technology has been used in the commercial potato French fries or chips industries [5].

Giteru, Oey and Ali [6] have recently reported that PEF could affect either the stability or the functional properties of biomacromolecules such as polysaccharides and proteins. Especially on polysaccharides, PEF could affect their microstructure, conformation, solubility, swelling effect, particle size, viscoelastic properties, structural transition and thermal stability [6]. Moreover, other studies have reported that PEF processing applied at an electric field strength up to 50 kV/cm can change the properties of starches dispersed in water, such as the structural properties and the digestibility of waxy rice starch [7]; the microstructure, thermal properties and viscosity of tapioca starch [8]; the microstructure and thermal properties of maize starch [9]; the thermal properties and microstructure of potato starch [10]; and the thermal properties and microstructure of corn starch [11]. So far, limited studies have been conducted to understand the fate of starch inside the potatoes after the tubers are PEF-treated. Starch is the major component in potato [12] that contributes to its nutritional quality [13,14]. Although food processing techniques [14,15], including boiling, cooling, reheating [16], conventional frying and air frying [17], have been shown to change the digestibility of starch, it is still not known whether PEF processing affects the inherent properties of starch in potatoes. In addition, the adoption of PEF technique in potato processing is fairly recent, compared with, say, the use of PEF in the processing of liquid foods, such as in juice extraction, bacterial inactivation in milk, etc. It is also known that consumer perception regarding the safety of PEF-processed foods is a key to their acceptance [18] and this perception is often influenced by information on the effect of PEF technology on the products themselves [19]. A similar phenomenon would potentially happen in the adoption of PEF in potato processing.

Therefore, the purpose of this study was to investigate the properties of starch in potatoes after being treated by PEF. The native state of starch isolated from potatoes after PEF treatment was examined using polarised light microscopy, combined with hot-stage optical microscopy. The gelatinisation behaviour, thermal stability and thermal properties of starch isolated from potatoes after PEF treatment were studied using thermogravimetric analysis (TGA) and differential scanning calorimetry (DSC). The susceptibility of the isolated starch towards heat stable α-amylase as well as the degree of starch hydrolysis during in vitro simulated human oral-gastro-intestinal digestion were studied. In this study, the effects of electric field strength and specific energy for PEF on these properties were investigated. In addition, the properties of any starch found in the PEF processing medium (SPM) (e.g., leached out of the potato due to cutting during sample preparation) were examined to allow a direct comparison with the properties of starch obtained from the same potatoes after being treated with PEF. To the authors’ knowledge, this research is the first work to study the properties of starch relevant to PEF processing of potatoes.

## 2. Materials and Methods

### 2.1. Chemicals and Reagents

Potassium iodate (KI), iodine (I_2_), potassium hydroxide (KOH), sodium hydroxide (NaOH) and hydrochloric acid (HCl), were purchased from Merck (Darmstadt, Germany). Sodium chloride (NaCl) was purchased from BDH Chemicals (Poole, UK). Potassium chloride (KCl) was purchased from Fisher Scientific (Norcross, GA, USA). Sodium bicarbonate (NaHCO_3_) was purchased from (Riedel-de Haën, Seelze, Germany). Heat stable α-amylase from *Bacillus licheniformis* (3000 U/mL)*,* amyloglucosidase from *Aspergillus niger* (3300 U/mL), glucose oxidase peroxidase (GOPOD) kit and D-glucose standard (1 mg/mL) were purchased from Megazyme (Wicklow, Ireland). Alpha amylase from *Aspergillus oryzae* (30 U/mg) and pancreatin from porcine pancreas (4 × USP) were purchased from Sigma (St. Louis, MO, USA). Pepsin was purchased from PanReac AppliChem (Barcelona, Spain). Porcine bile extract was purchased from Santa Cruz Biotechnology (Dallas, TX, USA). 

### 2.2. Preparation of Samples

**Figure 1:**
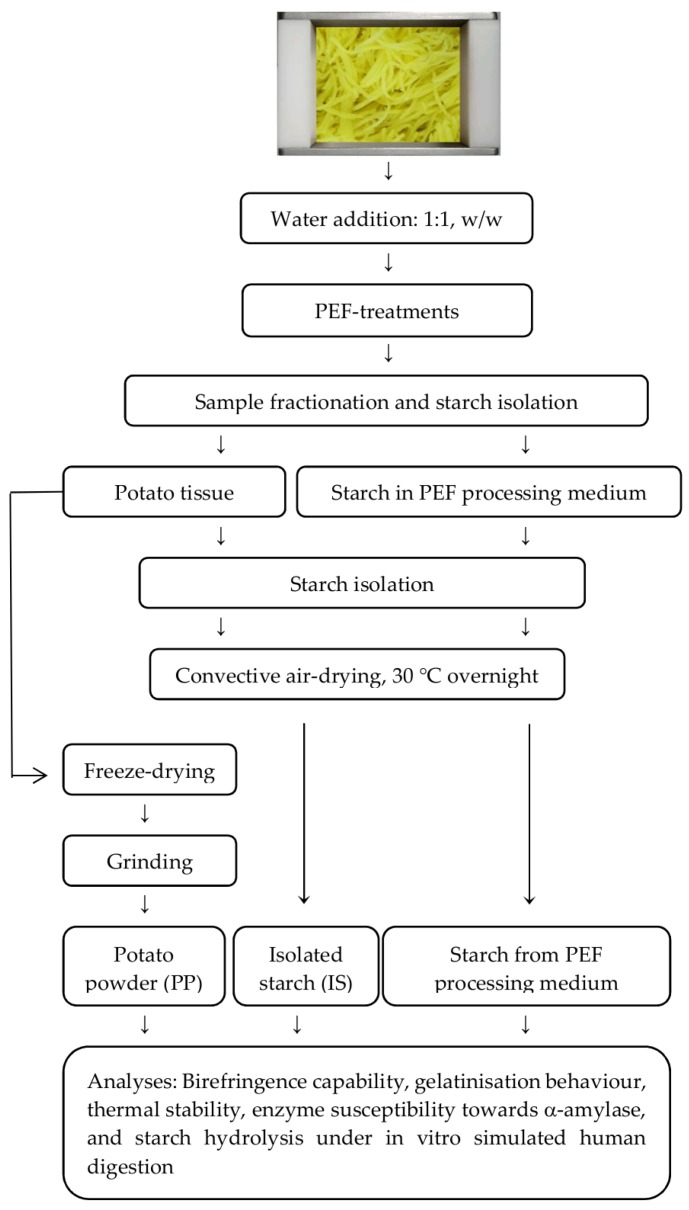
Schematic overview of the experimental design and sample collection followed by preparation.

A batch of potato tubers (*Solanum tuberosum* cv. Agria) harvested in August 2017 were obtained from Pyper’s Produce (Invercargill, New Zealand). In this study, the Agria cultivar was selected as a model system due to its high starch content. Upon arrival, tubers were sorted according to their weight, size and dimensions while any tubers with cuts, bruises or damage were discarded. Fifty potato tubers with uniform size and dimension were selected and randomly divided into five groups (10 tubers per group) which was later used as replicates. Each group of ten potatoes was peeled and shredded (2.79 mm × 2.79 mm) using an MX 260 food processor (Kenwood, Beijing, China) at medium speed. The shredded potato from 10 tubers were pooled together to attain a homogenous sample set and kept in an ice-water bath for no longer than 30 min. The samples were immediately treated with PEF (see Section 2.3) at different electric field strength and energy combinations (Table 1) following the experimental protocol presented in Figure 1. In total, five independent replicates were conducted for this study.

### 2.3. Pulsed Electric Fields Treatment

PEF processing was performed using an ELCRACK^®^ HVP 5 PEF system (German Institute of Food Technologies, Quakenbruck, Germany) in the batch treatment configuration. The PEF treatment chamber (total volume of 400 mL with dimensions of 100 mm length, 80 mm width and 50 mm depth), consisted of two parallel stainless-steel electrodes (80 mm electrode gap). For each treatment, 125 g of potato samples were placed inside the PEF treatment chamber and then submerged in 125 g of distilled water, which served as the PEF processing medium. The pulse shape generated by the PEF unit was monitored in real-time using an oscilloscope (UTD2042C, Uni-Trend Group Ltd., Dongguan, China). Output parameters, such as electric field strength (kV/cm), pulse voltage (kV), pulse current (A), pulse power (kW), pulse energy (J), total energy (kJ), pulse number (dimensionless) and pulse resistance (ohm), were recorded for each PEF run.

In this study, different field strengths, i.e., 0.5, 0.7, 0.9 and 1.1 kV/cm, accompanied with two specific energy input intensities averaged at 49.87 ± 0.54 and 153 ± 0.91 kJ/kg were used. The specific energy input was calculated using Equation (1):

(1) $$W_{specific}\;\left(kJ/kg\right)=\frac{V^{2}\cdot \left(n\cdot m\right)}{R\cdot W}$$

where *V* is the pulse voltage (kV), *n* is the pulse number (dimensionless), *m* is the pulse width (µs), *R* is the pulse resistance (ohm) and *W* is the total weight of the sample and PEF processing medium.

All tested PEF process conditions were achieved by applying a 20 µs pulse width at 100 Hz frequency with the pulse number ranging from 900–6250. Each PEF treatment was carried out in five replicates with each replicate utilising shredded potatoes from 10 tubers. The process of treating potatoes with PEF for each run was standardised and took no more than 3 min. For every PEF run, the untreated sample (i.e., potato samples without PEF treatment) was prepared by soaking the potato samples in distilled water at a ratio of 1:1 for 3 min and afterwards the sample was handled as described in Figure 1.

To test whether PEF causes changes in cell permeability, both conductivity and temperature of the processing medium were first measured just before and immediately after PEF treatment using a CyberScanCON11 (Eutech Instruments, Singapore, Singapore) hand-held conductivity meter to have an indication of increased ion leakage due to PEF treatment. Subsequently, the PEF processing medium was separated from the potato sample using a kitchen sieve and transferred into plastic containers (1000 mL volume). Secondly, the colour of the PEF processing medium was immediately measured on the tristimulus colour combination L*a*b* scale using a MiniScan XEPlus 45/0-L colorimeter (Hunterlab, Reston, VA, USA) in triplicate. The tristimulus colour combination of L*a*b* were then converted into a browning index (BI) using Equations (2) and (3):

(2) $$BI=\frac{100\left(X-0.31\right)}{0.17}$$

with:

(3) $$X=\frac{a+1.75L}{5.645L+a-3.012b}$$

### 2.4. Isolation of Starch after PEF Treatment

Immediately after PEF treatment, both untreated and PEF-treated potato samples were separated from the processing medium using a kitchen sieve. Potato samples (approximately 125 g) and the processing medium were transferred into separate plastic containers for starch isolation as described below.

Potato starch was isolated from 100 g potato samples according to [20] with modifications. Potato samples, either untreated or PEF-treated, were placed into a plastic container containing 300 mL distilled water and, afterwards, the samples were gently macerated by hand for 1 min. The mixture of potato and distilled water was then filtered using a kitchen sieve and the filtrate was collected. The maceration process on the potato samples was repeated with another fresh 300 mL of distilled water. The final filtrate was combined and kept at room temperature for 2 h to allow the starch to settle to the bottom. After 2 h, the water layer on the top of the starch suspension was discarded and replaced with fresh distilled water. This step was repeated twice until clear water was obtained. The starch sediments were then oven dried (Eurotherm 3216, Steridium, Queenstown, Australia) at 30 °C overnight or longer until the starch was completely dry (indicated by no further weight loss). The dried powder, referred as “isolated starch” (later coded as “IS”), was transferred into 1.5 mL tubes and stored in a desiccator filled with silica gel at ambient temperature (17–20 °C) until further analysis. 

PEF processing medium (approximately 125 mL) was transferred into a plastic container followed by the addition of 300 mL distilled water. The mixture was kept at room temperature for 2 h to allow the starch to settle. After 2 h, the water layer on the top of starch suspension was discarded and replaced with fresh distilled water. This step was repeated twice until clear water was finally achieved. The starch sediments were then oven dried (Steridium with Eurotherm 3216 controller) at 30 °C overnight or longer until the starch was completely dry (indicated by no further weight loss). The dried powder obtained was referred as “starch from PEF processing medium” (later coded as “SPM”). The sample was kept in 1.5 mL tubes and stored in a desiccator filled with silica gel at ambient temperature (17–20 °C) until analysis.

The remaining potato samples (approximately 25 g) was transferred into plastic bags and kept frozen at −20 °C (Fisher and Paykel, Auckland, New Zealand) for no more than 2 weeks, followed by freeze drying (Labconco Freezone, Kansas City, MO, USA). Subsequently, the freeze-dried samples were homogenised into powder form (thereafter referred as “potato powder or PP” sample) using a laboratory blender (32BL80 Waring, Torrington, CT, USA) for 10 s at high speed. They were sealed tightly inside polypropylene vials under ambient temperature (17–20 °C) until analysis.

### 2.5. Study on the Birefringence Capability of Starch Granule after PEF Treatment

The native form of starch typically exhibiting birefringence capability is associated with the crystalline structure of starch. The birefringence capability of starch isolated from PEF-treated potatoes was studied by mean of light microscopy and polarised microscopy. Starch suspension was prepared by gentle mixing of 2 mg dried sample and 250 µL deionised water. A drop of starch dispersion was transferred onto a glass slide (LabServ, Waltham, MA, USA) and a small amount of Lugol’s iodine dye (5% (*w/v*) potassium iodate and 0.5% (*w/v*) iodine at a ratio of 1:1) was added. After the cover slip was placed, the specimen was observed under a BX-50 microscope (Olympus, Tokyo, Japan) with a polariser (U-POT U-P110 model, Olympus, Tokyo, Japan) under a magnification of 400×. The observations under the microscope were captured using a Camedia C4040 Zoom digital camera (Olympus, Tokyo, Japan). The images were saved as TIFF files and later standardised for their brightness using Windows Photos (Microsoft, Redmond, WA, USA).

### 2.6. Study on the Gelatinisation Behaviour of Starch Using Hot-Stage Microscopy

A light microscope, Motic BA300Pol (OPTIKA SrL, Ponteranica, Italy), complete with polariser, at a magnification of 200× was used. Starch dispersions (2 mg/250 µL deionised water) were prepared and then transferred onto glass slides with cavities, covered with a coverslip and put onto a hot stage (FP82HT model, Mettler Toledo, Columbus, OH, USA) connected with a Mettler Toledo FP90 central processor to control the heating setting of the hot stage. The specimen was heated from 30–80 °C at a rate of 5 °C/min. Live pictures of the specimen were automatically recorded every 15 s which was equal to a temperature increment of 1.25 °C. The pictures were captured using a Nikon Optiphot PFX microscope camera (Nikon, Tokyo, Japan) and the files were saved as BMP files with Image-Pro Plus version 2.7 software (Media Cybernatics Inc., Rockville, MD, USA). The captured images were resized using Windows Photos (Microsoft).

### 2.7. Study of the Thermal Stability of Starch

Thermogravimetric analysis (TGA) was used to determine the moisture content and the thermal stability of the starch-containing dried samples. Dried sample, about 10 mg, were transferred onto a 100 µL platinum TGA pan (TA Instruments, New Castle, DE, USA). Subsequently, the sample was heated from room temperature to 400 °C inside the TGA 550 unit (TA Instruments). The TGA operation and the data analysis to determine the weight loss and derivative weight loss of the sample during heating were performed using TRIOS software V4.3 (TA Instruments).

### 2.8. Study on the Gelatinisation Temperature of Starch Using Differential Scanning Calorimetry

Starch-containing samples were weighed closed to 3.0 ± 0.5 mg (in dry basis as predefined using TGA) on Tzero DSC pan (TA Instruments). Adequate amount of distilled water was added to achieve 70% moisture in the sample and the pan was sealed tightly using Tzero hermetic lid with the assistance of a Tzero press with blue die set (TA Instruments). Samples were then allowed to equilibrate to room temperature for 1.5 h prior to analysis in a DSC 250 unit (TA instruments) calibrated with indium (purity >99.9%) and heated from 20–100 °C at a 10 °C/min heating rate. The DSC operation and analysis on the temperatures at which the starch underwent phase transition during heating, i.e., temperature of onset gelatinisation *To*, temperature of peak gelatinisation *Tp*, temperature of conclusion gelatinisation *Tc*, range of gelatinisation temperature *R* and enthalpy change of gelatinisation *∆H* were performed using TRIOS software V4.3 (TA Instruments).

### 2.9. Study on the Susceptibility of Starch towards Enzymes

#### 2.9.1. Starch Susceptibility towards Heat Stable α-Amylase

Twenty milligrams of sample was transferred into a 50 mL falcon tube, added with 980 µL distilled water and equilibrated to room temperature for 10 min. The dispersion was then vortexed and placed on a magnetic stirrer followed by the addition of 1 mL KOH (2M) to dissolve any resistant starch in the sample. The mixture was allowed to stir for 20 min in an ice water bath over the magnetic stirrer. Another 4 mL of sodium acetate buffer (1.2 M; pH 3.8) was then added to neutralise the pH of the mixture. Then, 50 µL of heat stable α-amylase and 50 µL amyloglucosidase were added, followed by incubation at 50 °C in a water bath for 1 h with intermittent vortexing at every 10 min to hydrolyse insoluble starch into soluble branched and dextrin and to hydrolyse the dextrin into D-glucose. After that, the mixture volume was brought up to 40 mL with deionised water, vortexed and centrifuged (Beckman GPR, Beckman, Indianapolis, IN, USA) with an acceleration of 1613× *g* for 10 min. Fifty microliters of the supernatant was then added with 1.5 mL GOPOD reagent and heated at 50 °C for 20 min. Afterwards, the absorbance was measured at wavelength of λ = 510 nm and temperature of 20 °C using a UV–VIS spectrophotometer (Ultraspec 3300 Pro, Amersham Biosciences, Amersham, UK) with D-glucose (1 mg/mL) solution as the external standard solution. Values were expressed as percent (*w/w*) hydrolysed starch per sample using a conversion factor of 0.9, which is generally calculated from the molecular weight of starch monomer/molecular weight of glucose (162/180 = 0.9) [21,22].

#### 2.9.2. Starch Hydrolysis under In Vitro Simulated Human Digestion System

In vitro simulated human digestion of starch-containing dried samples consisted of three phases, namely oral, gastric and small intestinal phases, was carried out as outlined in [23] with modifications. Forty milligrams of sample were transferred into glass vials and added with 1960 µL deionised water. Afterwards the mixture was mixed gently with a vortex and equilibrated for 10 min at 20 °C. Subsequently, 2 mL simulated salivary fluid containing NaCl (2 mM), KCl (2 mM) and NaHCO_3_ (25 mM) and 1 mL α-amylase from *Aspergillus oryzae* (12.5 mg/mL) was added. The mixture was then incubated for 5 min at 37 °C (LabServ, Contherm Scientific Ltd., Wellington, New Zealand) with shaking at 55 strokes per min using a rocking shaker (DLAB Scientific Inc., Beijing, China). Incubation with shaking was carried out for the next 2 h after the pH was adjusted to about 3 with HCl (1 M) and 4 mL simulated gastric juice (1 mM HCl) containing 40 mg/mL pepsin, 151 mM NaCl and 28 mM KCl was added. Upon completion of gastric digestion, the pH was adjusted to pH 7 using NaOH (1 M) and simulated intestinal fluid of NaHCO_3_ (0.1 M) containing pancreatin from porcine pancreas (10 mg/mL) and bile extract (8.45 mg/mL) was added and incubated with shaking for another 2 h. During the entire course of simulated intestinal digestion, 1 mL digest was withdrawn at time 0, 20, 60, 90 and 120 min and immediately transferred into a 15 mL tube containing 5 mL ethanol (80%). Individual digests were then centrifuged with an acceleration of 1613 g for 10 min at 5 °C and the entire supernatant was used for further analysis. 

The supernatant of digested samples was added with 50 µL amyloglucosidase (3300 U/mL) and afterwards incubated at 50 °C for 1 h with an intermittent vortexing for every 10 min. Fifty microliters from the mixture were added with 1.5 mL GOPOD reagent, incubated at 50 °C for 20 min, and the absorbance was measured wavelength of λ = 510 nm and temperature of 20 °C using a UV–VIS spectrophotometer (Ultraspec 3300 Pro, Amersham Biosciences). D-glucose solution (1 mg/mL) was used as the external standard solution. Values were expressed as mean values with standard deviations of mg glucose per mL digest.

### 2.10. Statistical Data Analysis

In this study, results are expressed as the mean ± standard deviation of five independent treatment replicates. Statistical analyses were performed with IBM SPSS Statistics version 24 (IBM Corporation, New York, NY, USA) using one-way analysis of variance (ANOVA) followed by the Tukey’s HSD post hoc test. Differences between the means were considered significant when *p* < 0.05. Independent samples *t*-test was used to assess the significant differences of the thermal stability, gelatinisation temperature, α-amylase susceptibility and the degree of starch digestibility between untreated and PEF-treated samples.

## 3. Results and Discussion

### 3.1. Monitoring the Impact of PEF Treatment on Potatoes

In this study, the changes in the temperature and the conductivity of the product were evaluated to indicate whether all the PEF conditions applied to the potato samples led to cellular damage [1,24,25]. Table 1 clearly showed a considerable increment in the temperature and product conductivity for all the PEF treatment conditions applied. The increase in temperature after PEF treatment at specific energies of 50 and 150 kJ/kg was averaged at 6.07 °C and 14.61 °C, respectively. The temperature increase was due to the external energy generated from the PEF treatment while the increase in product conductivity were at least 0.44 to 0.91 mS/cm higher after PEF treatments than untreated samples (i.e., 0.25 mS/cm, see Table 1) indicating the leaching of ionic species inside the cells from the minerals ions and other soluble solids into the processing medium. The same ionic species were presumably freed during preparation of untreated (No PEF) samples but in the lower concentration which led to an increase in conductivity at a lower value, 0.25 mS/cm.

Browning index in the PEF processing medium was also considered in the present study owing to the possibility of enzymatic reaction occurring between polyphenoloxidase and phenolic compounds released from their cell localisation inside potato tissues [25] into the PEF processing medium after PEF treatment. Result showed that the browning index in the processing medium increased up to 294.73 ± 15.97 after the PEF treatments compared to that of untreated samples (i.e., 218.30 ± 33.80; see Table 1) indicating the leaching of phenolic compounds and polyphenoloxidase. It is important to note that phenolic compounds in potato tissues are localised in the vacuole while polyphenol oxidases are accumulated in the plastids [26]. Therefore, it is likely that the applied PEF treatments had effectively resulted in cellular damage, i.e., microstructural modification due to the formation of cell pores that facilitated the substrate-enzyme interaction in the processing medium. As a consequence, this triggered the enzymatic browning reaction, producing reddish-brown *o*-quinones compounds that contribute towards the browning of the PEF processing medium.

### 3.2. Comparison on the Birefringence Capacity of Potato Starch Granules after PEF Treatment

**Figure 2:**
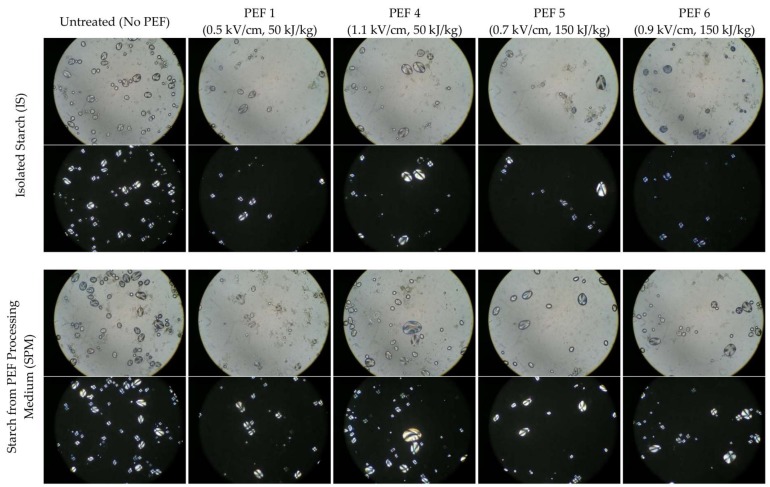
Selected light (**top**) and polarised (**bottom**) micrographs at a magnification of 400× of isolated starch (IS) and starch from PEF processing medium (SPM) of untreated and PEF-treated potatoes.

Starch granules have a semi-crystalline structure [27] which exhibits birefringence with a “Maltese cross” feature under a polarised microscope. The starch birefringence has been used as a good indicator to assess the native state of starch [28,29,30]. Figure 2 presents the microscopy images of starch granules isolated from potatoes after being treated with PEF (i.e., IS samples) and starch found in the PEF processing medium (i.e., SPM samples) using visible and polarised microscopes. 

Under polarised microscopy, both starch granules isolated from potatoes (IS) without PEF treatment and from processing medium (SPM) exhibit birefringence with the typical “Maltese cross” feature. In comparison, the same birefringence pattern distinct for native granules was observed for both IS and SPM samples isolated from potatoes PEF-treated at an electric field strength of 0.5 kV/cm with specific energies of 50 kJ/kg (PEF 1) in conjunction with PEF at 0.7 kV/cm with a specific energy of 150 kJ/kg (PEF 5) and also when the electric field strength was increased from 0.5 to 1.1 kV/cm and from 0.7 to 0.9 kV/cm, respectively, at constant specific energies of 50 (PEF 4) and 150 kJ/kg (PEF 5). This study clearly showed that PEF treatment at the processing intensities used in the current study did not influence the molecular crystallinity of starch granules from their native state. 

### 3.3. Understanding the Gelatinisation Behaviour of Starch Isolated from PEF-Treated Potatoes

One of the unique properties of starch is its ability to undergo gelatinisation under sufficient heat and moisture [28]. The starch granules gradually swell with increasing temperature, followed by a loss of crystallinity and the birefringence of the starch [31]. 

**Figure 3:**
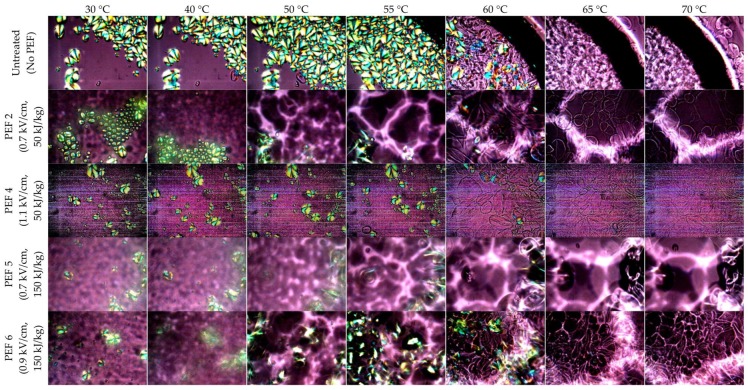
Selected photomicrographs, at a magnification of 200×, of isolated starches from untreated and PEF-treated potatoes, dispersed in water and heated from 30 to 70 °C at a rate of 5 °C/min.

Figure 3 presents the selected microscopy images of starch dispersion being heated at different temperatures. A representative video (Video S1) of gelatinisation behaviour of starch is attached as a supplementary material. Being heated at temperatures of 30, 40, 50 and 55 °C, all starch granules from untreated potato samples continuously showed the existence of birefringence. At 60 °C, most of the starch granules started to lose their birefringence and the birefringence was completely lost at 65 °C. For starch isolated from potato samples treated with PEF at specific energies of 50 (PEF 2) and 150 kJ/kg (PEF 5) with an electric field strength of 0.7 kV/cm, it was found that birefringence of starch was lost extensively at 60 °C, compared with starch from untreated samples that lost birefringence at 65 °C. With respect to PEF treatments on potato samples involving electric field strengths of 1.1 kV/cm and 0.9 kV/cm at specific energies of 50 (PEF 4) and 150 kJ/kg (PEF 6), respectively, there was no obvious indication of the loss of starch birefringence at temperature lower than 60 °C. Likewise, a complete loss of birefringence occurred at a similar temperature as all other starches, i.e., around 65 °C for both untreated and PEF-treated samples. Overall, it was clear that starch isolated from PEF-treated potatoes were retaining the same gelatinisation behaviour as starch from untreated potatoes. It is important to note that it is rather challenging to predict precisely the starting gelatinisation temperatures for the starch under hot-stage microscopy and, hence, the differential scanning calorimetry (DSC) method (Section 3.5), being a more reliable approach, was used to exhibit the onset, peak and conclusion temperatures of the starch gelatinisation process.

### 3.4. Effect of PEF on the Thermal Stability of Potato Starch Granules

**Figure 4:**
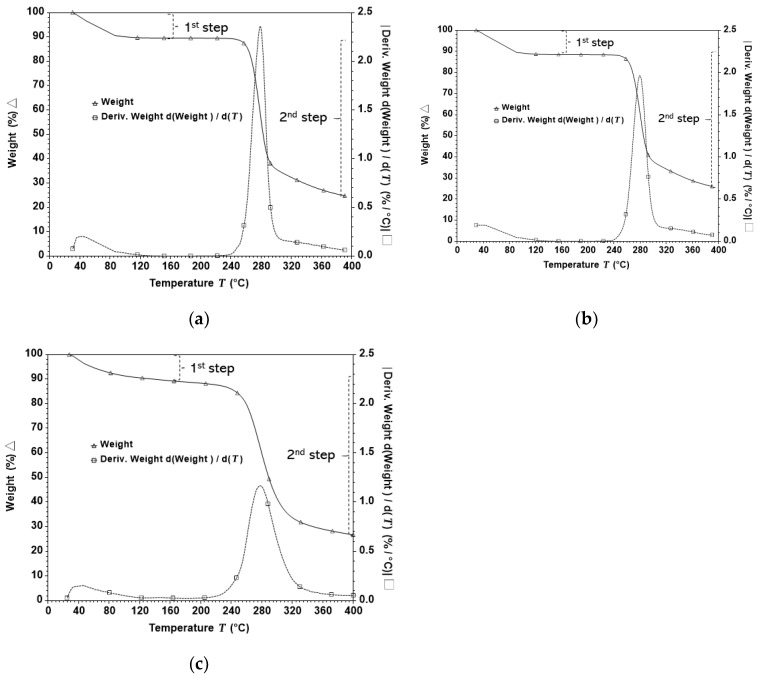
Typical TGA thermograms and their derivative weight of isolated starch (**a**), starch from processing medium (**b**), and potato powder (**c**) from untreated and PEF-treated potatoes. The thermograms are similar for each fraction among PEF treatments analysed in triplicate.

Figure 4 presents the typical curves of weight (%) and its derivative over the temperature (%/°C) of thermogravimetric analysis (TGA) of starch isolated from potatoes (IS) and starch leached out in the processing medium (SPM). In this study, potato powder (PP) was used as a reference to represent the original sources of the isolated starch.

In this study, these three types of samples exhibited similar TGA profiles, which are characterised by two steps of weight loss occurring at similar temperatures. The first step represented the loss of moisture, occurred at about 30 °C and continued until the weight remained constant at about 235 °C. However, compared with those of IS and SPM samples, the initial weight loss on the PP sample occurred slower prior to achieving the constant weight. This indicated that the moisture in the PP sample was bound at a stronger level due to the presence of potato tissue which highly contains water binding compounds, such as pectin, amounting up to 52% of potato cell walls [32]. 

The second step of weight loss, which occurred at about 260 °C, represents the decomposition of starch polymer resulting in the formation of CO, CO_2_ and H_2_O due to the degradation C-O and C-C bonds [33]. The most intensive decomposition of polymer was found to occur at a similar temperature across IS, SPM and PP samples, indicating that these three types of samples have polymers with the same properties from where those potato starch granules were extracted. The most intensive decomposition temperatures, indicated as *Tpdw* in Table 2, were 278.35 ± 1.21 °C, 279.78 ± 1.55 °C, and 276.78 ± 3.77 °C, respectively, for IS, SPM and PP.

The typical TGA curve in the current study was consistent with the work carried out on corn starch with two steps of weight losses [34]. However, on the aforementioned study, the *Tpdw* was found to be about 300 °C, similar to that of maize starch [9]. Therefore, *Tpdw* can be directly and uniquely attributed to the type of crystalline structure of the starch. Starch from cereals is characterised by the A-type crystalline structure while starch granules from tubers are usually characterised by the B-type of crystalline structure [35]. A similar TGA profile was also found in another potato starch study [36]. Furthermore, the slower weight loss occurring at the first step of weight loss on Agria cultivar in the current study is similar with those of Agata and IAPAR Cristina cultivars [37]. However, the latter study showed higher *Tpdw*, i.e., 300 °C and 298 °C, respectively, for Agata and IAPAR Cristina. Thus, thermal stability of potato can also be dependent on the biological origins of samples.

The IS, SPM and PP samples obtained from potatoes treated with PEF at increasing specific energies from 50–150 kJ/kg, as well as at increasing electric field strengths from 0.5–1.1 kV/cm were found to have negligible influence on the *Tpdw*. In other words, the *Tpdw* values for all starch samples obtained from PEF-treated potatoes were not significantly different from their untreated counterparts. This finding is consistent with the work of Han with his co-workers [9] who reported that thermal stability of maize starch remained unchanged (*Tpdw* at about 300 °C) even after PEF treatment at high intensity electric field strengths between 30 and 50 kV/cm. 

### 3.5. Study on the Effect of PEF on the Gelatinisation Temperature of Potato Starch Granules

**Figure 5:**
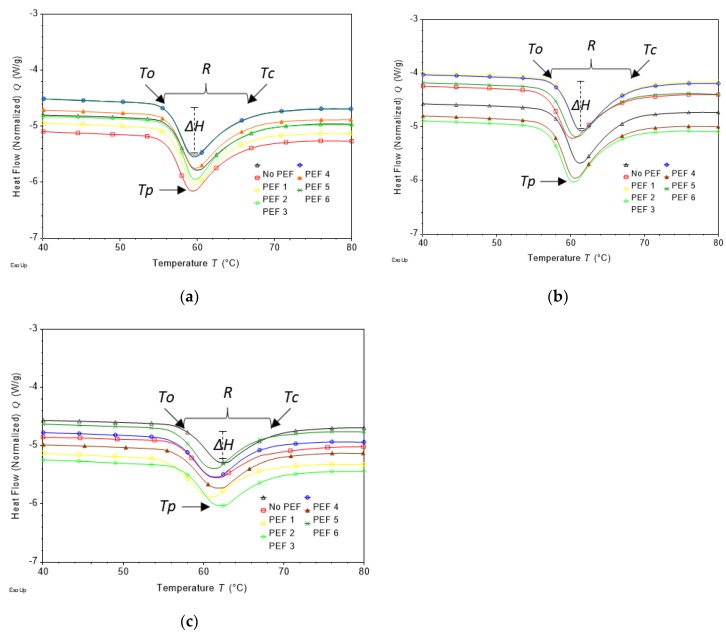
DSC thermograms of isolated starch (**a**), starch from processing medium (**b**), and potato powder (**c**) from untreated (No PEF) and PEF-treated potatoes at 50 kJ/kg specific energy with field strengths (kV/cm) of 0.5 (PEF 1), 0.7 (PEF 2), 0.9 (PEF3), 1.1 (PEF 4) and at 150 kJ specific energy with field strength (kV/cm) of 0.7 (PEF 5) and 0.9 (PEF 6). *To*: temperature of onset gelatinisation, *Tp*: temperature of peak gelatinisation, *Tc*: temperature of conclusion gelatinisation, *R*: gelatinisation temperature range, *ΔH*: enthalpy change of gelatinisation.

Temperatures at the onset (*To*), peak (*Tp*) and concluding stage of gelatinisation (*Tc*), as well as the temperature range (*R = Tc–To*) and enthalpy change during gelatinisation (*ΔH*), are important parameters in investigating the gelatinisation behaviour of starch. These parameters can be obtained using the method of differential scanning calorimetry (DSC) [20,30,38,39]. Figure 5 presents the DSC thermograms for IS, SPM and PP with the corresponding temperature values are presented in Table 2. In the same manner as TGA assay, potato powder (PP) was used as a reference in this DSC assay to represent the original sources of the isolated starch. The IS, SPM, and PP samples from untreated potatoes gelatinised at *To* of 56.67–57.69 °C followed by *Tp* between 59.81 and 62.08 °C, and finally reached *Tc* between 64.83 and 67.75 °C. The corresponding temperature range (R) was narrow for IS samples (8.16 ± 1.87 °C), followed by SPM samples at 9.39 ± 0.20 °C and the widest for PP at 10.30 ± 0.47 °C. Overall, it was demonstrated that IS, SPM and PP samples from untreated potatoes shared some similarities in the gelatinisation temperatures, but DSC thermograms also showed that PP samples experienced the lowest *ΔH* (26.26 ± 0.23 J/g) compared to IS (33.37 ± 7.09 J/g) and SPM (37.32 ± 1.97 J/g) samples. 

The present study found that starches isolated from any PEF-treated potatoes (IS), either at increasing electric field strengths up to 1.1 kV/cm and increasing specific energies up to 150 kJ/kg, were gelatinised in a similar matter as starch isolated from untreated potatoes. It indicated that starch in potato tissue was not prone to PEF treatment probably due to its location in potato tissue. The PEF energy delivered to the potato tissue lead to pore formation on the cell membrane as indicated by the increase in conductivity and browning index, but it was not sufficient to cause the change in the starch structure.

With respect to the starch from PEF processing medium (SPM), those samples derived from PEF-treated potatoes were found to share similar values for gelatinisation temperatures of *To*, *Tp* and *Tc*. However, there was a significant difference in the gelatinisation temperature range (R) among the SPM samples owing to the intensity of PEF initially applied to the potato samples. In particular, SPM samples from potatoes after PEF treatment at higher specific energies of 150 kJ/kg (PEF 5 and PEF 6) were found to have a narrower gelatinisation temperature range compared to SPM sample from untreated potatoes, i.e., R decreased from 9.39 to 7.98 °C. Since the range of gelatinisation temperature is inversely proportional to the degree of cohesion between crystallites of starch [40], a narrow range of gelatinisation temperature reflects a stronger cohesion between crystallites, particularly after PEF treatment at high specific energy. Moreover, a narrow gelatinisation temperature range exhibited by SPM samples at high-energy PEF treatment could be due to no potato tissue was present to protect the starch granules from the PEF energy. Hence, the starch was more prone to the PEF treatment. It is important to note that the starch found in the processing medium is generally represented by starch granules available at the surface of potato tissue which were washed out into the PEF processing medium during the process. 

On PP samples, apparent changes were found on PEF-treated samples such as in *Tp* of PEF 1, PEF 2, PEF 6 and *ΔH* of all PEF treatments. These changes were presumably associated with the variation in the amount of starch in PP sample during DSC analysis considering that the sample size is limited to about 2.45 mg dry sample whereas, in fact, a lesser amount of starch in the sample matters in lowering the peak height of *Tpdw* in the thermal stability of PP sample compared with those of IS and SPM samples, as shown by the finding in TGA analysis.

The effect of PEF on the narrower temperature range of gelatinisation (*R*) of SPM sample in the current study was different from the work carried out on 8% (*w/w*) potato starch in water dispersion and PEF-treated at intensities up to 50 kV/cm [10]. In the Han and co-workers study [10] the temperature range of gelatinisation was slightly broadened with increasing field strength from 30 to 50 kV/cm indicating less structuring of the resulted starch granule after PEF treatment. Furthermore, the previous study did not mention whether specific energy was also important in the change of gelatinisation temperature range. The different phenomenon observed in this study and that of Han and co-workers [10] could also be due to the differences in the potato cultivar and PEF processing parameters used. 

### 3.6. Susceptibility of Starch Granules from PEF-Treated Potatoes towards Heat Stable α-Amylase

The susceptibility of starch to heat stable α-amylase is an important property of starch [41]. This indicates the starch damage [42] occurring in the development of porous starch granule [43] and damage found on the starch due to processing [44] which influence starch functionalities. Unlike corn starch and starch from cereal sources, potato starch is described as a very large and smooth granule [45] with a relatively well-ordered and dense structure. For this reason, potato granules are considered to be relatively resistant to hydrolytic enzymes such as amyloglucosidase and α-amylase [12,46]. As was done for the TGA and DSC assays, potato powder (PP) was used as a reference in the enzyme susceptibility assay to represent the original sources of the isolated starch.

Table 3 presents the susceptibility of IS, SPM, and PP from PEF-treated potatoes and untreated samples expressed as total hydrolysed starch. The total hydrolysed starch found in the current study were on average 68.62% ± 4.08%, 73.09% ± 2.65%, and 62.83% ± 6.16%, respectively, for IS, SPM and PP from untreated potatoes. These values are comparable with the hydrolysed starch reported in other potato cultivars which range from about 68–73% [47]. Moreover, PP samples, regardless of the level of PEF-treatment, consistently showed a lower enzyme susceptibility towards heat stable α-amylase compared to their corresponding IS and SPM samples. This could be due to a lower starch content available in the PP samples. Results from TGA (Table 2) further support this assumption since it was found that the height of *Tpdw* peak of the PP samples was typically lower than IS and SPM samples. 

With respect to PP samples, it was clear that any PEF treatments applied on the potatoes did not significantly impact their enzyme susceptibility towards heat stable α-amylase. Likewise, for starch inside the potatoes (IS samples), this study showed that PEF treatments led to no statistically significant effect on their susceptibility towards heat stable α-amylase. However, it was interesting to find that IS samples from PEF-treated potatoes at an electric field strength of 0.7 kV/cm regardless of the specific energy applied (PEF 2 and PEF 5) consistently exhibited higher susceptibility towards heat stable α-amylase compared with those of IS samples from untreated samples and those from potatoes treated at other electric field strengths. Such finding indicated that 0.7 kV/cm could be an optimum electric field strength to be applied on potatoes in order to improve the susceptibility of starch in the PEF-treated potatoes towards heat stable α-amylase leading to a better digestibility.

With respect to the starch found in the processing medium (SPM samples), the impact of PEF treatment on their enzyme susceptibility was also not statistically different. However, it is important to note that SPM samples from potatoes treated at an electric field strength of 0.9 kV/cm combined with a specific energy of 50 (PEF 3) showed a significant improvement in the susceptibility towards heat stable α-amylase compared to SPM sample from untreated potatoes. Another interesting finding was that when applying a higher intensity of specific energy (from 50 to 150 kJ/kg) on potatoes at either electric field strength of 0.7 or 0.9 kV/cm resulted in the corresponding SPM samples (PEF 2 vs. PEF 5, PEF 3 vs. PEF 6) to be more susceptible towards heat stable α-amylase. This phenomenon related to the improved enzyme susceptibility as the result of the application of increasing specific energy for potatoes was only observed for SPM starches. IS or PP starches could not be associated with the disruption of starch granules and the crystalline structure responsible for gelatinisation remained unchanged. An enhancement effect on enzyme susceptibility with increasing specific energy for the SPM was consistent with the structural disruption found for PEF-treated potato starch dispersed in water [10,48].

### 3.7. Enzyme Susceptibility of Starch from PEF-Treated Potatoes under In Vitro Simulated Human Digestion Condition

The in vitro simulated human digestion assay was used to assess the susceptibility of the starches in potatoes after being treated with PEF towards digestive enzymes. Table 4 summarises the glucose release per volume digest (mg/mL) of simulated human intestine phase at different digestion period i.e., 0, 20, 60, 90 and 120 min from IS and SPM isolated from PEF-treated potatoes and untreated control, compared to its reference, PP samples. 

This study found that PEF at all treatments did not significantly change the in vitro simulated digestibility of IS but did pose some major influence on selected SPM and PP samples. After undergoing 120 min of in vitro simulated human digestion during the intestinal phase, it was found that SPM samples from potatoes treated with PEF, particularly PEF 4 and 6, showed a slight reduction in starch digestibility and a prominent reduction was found for that of PEF 4 treatment (i.e., a lower amount of glucose released compared to SPM samples from untreated potatoes). With respect to PP samples, the most distinct difference in the starch digestibility was found after these samples had undergone 90 min of in vitro simulated human digestion during the intestinal phase. PP samples from potatoes treated with PEF, particularly PEF 1 and 6, showed a considerable improvement in starch digestibility (i.e., a higher amount of glucose released compared to PP samples from untreated potatoes).

Some general findings can be seen in the starch digestibility. PEF-treated samples of SPM tend to consistently decrease in starch digestibility. It is presumably due to the disruption in starch structure as indicated by the change in narrowed temperature range of gelatinisation R, as shown by DSC analysis. PEF treated samples of PP tend to be consistently higher in starch digestibility than those of non-PEF treated materials. This indicated that potato tissue was more prone than starch to PEF treatment as already proven by the fact that no change was found in starch properties of IS samples after PEF treatment. Consequently, pores on cell membranes were formed after PEF treatment [1] leading to facilitation of enzyme diffusion to reach the starch. Furthermore, the trend of PEF effect on digestibility of SPM and PP samples was consistently found after the most intensive PEF (PEF 6) which shows the most extreme change in starch digestibility compared with their untreated counterparts.

The finding in starch digestibility of PEF-treated potato gave an indication that concern to nutritional attributes and safety of starch from PEF treated potato is unwarranted, as already shown that starch in potato remained unchanged after PEF treatment. The increase in starch digestibility of potato (powder) is an additional impact of modification in microstructure of solid plant food after PEF treatment [1] from other impacts that has been previously studied, such as the reduction of cutting force for potato [2] and sweet potato [3], the oil uptake during frying and retaining the textural properties [4]. Furthermore, PEF treatment at a proper condition without leading to excessive external energy could potentially be used on starch to decrease its digestibility.

Regarding the nutritional status of starch, a decrease in starch digestibility is considered as healthy for some cohorts of consumers. In this context, starch digestibility [22] is divided into (a) rapidly digestible starch (RDS): starch digested within 20 min; (b) slowly digestible starch (SDS): starch digested between 20 and 120 min; and (c) resistant starch (RS): starch digested after over 120 min. The health benefit of decreased digestibility of starch can be attributed either to SDS [49] or RS [50]. SDS is considered as healthy to lower the risk of drastic increase of postprandial blood sugar while RS is considered as healthy to feed the colon microbiome leading to colonic health of the host [50]. Thus, RS has been considered as prebiotic [51] and adopted as a functional ingredient [52]. 

Therefore, a decrease in digestibility of starch is an intended outcome of food processing to produce resistant starch [53]. Some processing techniques that have been used to lower starch digestibility are gamma irradiation of corn starch [54], high pressure treatment of starch from wheat, tapioca, potato, corn, and waxy corn [55], heat moisture treatment of mung beans [56] and rice starch [57], dual autoclaving-retrogradation of rice starch [58] and annealing of common buckwheat starch [59]. The outcome of the present study shows that PEF can be considered as a technology that contributes to lowering starch resistance.

## 4. Conclusions

This study confirmed that the starch inside potatoes after being treated with PEF remained in its native state as indicated by the presence of birefringence properties under a polarised microscope. The thermal stability, gelatinisation behaviour, susceptibility of starch in potato to heat-stable α-amylase and digestive enzymes under in vitro simulated human digestion conditions remained unchanged after PEF treatment. However, starch on the surface of potato (that leached into the medium, as SPM) apparently was found to be more prone to PEF treatment as indicated by a narrow range of the gelatinisation temperature especially after PEF treatment at 150 kJ/kg, leading to less digestible starch. Since PEF processing did not change the properties of starch in potatoes as shown in this study it is suggested that the phenomena previously reported in the literature, such as reduced processing intensities for frying or changes in sensory properties after frying, is driven more by other factors, such as structural changes of the potato tissues, and not by modification of potato starch granules. 

## Figures and Tables

**Table 1:** Summary of PEF treatment parameters on potato and the treatment impact on the changes in electrical conductivity, temperature and browning index of the PEF processing medium.

PEF (kV/cm, kJ/kg)	Electric Field Strength (kV/cm)	Specific Energy Input (kJ/kg)	Conductivity Increase (mS/cm) *	Temperature Increase (°C) **	Browning Index
Untreated (No PEF)	0.00	0.00	0.25 ± 0.16 ^b^	1.56 ± 2.32 ^c^	218.30 ± 33.80 ^c^
PEF 1 (0.5, 50)	0.50	50.48 ± 1.10	0.61 ± 0.17 ^ab^	6.42 ± 1.25 ^b^	279.74 ± 24.23 ^ab^
PEF 2 (0.7, 50)	0.70	49.25 ± 0.44	0.44 ± 0.33 ^b^	5.90 ± 0.53 ^b^	268.36 ± 34.47 ^ab^
PEF 3 (0.9, 50)	0.90	49.63 ± 0.31	0.54 ± 0.08 ^ab^	6.08 ± 0.99 ^b^	294.73 ± 15.97 ^a^
PEF 4 (1.1, 50)	1.10	50.10 ± 0.39	0.53 ± 0.07 ^ab^	5.86 ± 0.39 ^b^	269.13 ± 27.05 ^ab^
PEF 5 (0.7, 150)	0.70	151.81 ± 1.72	0.91 ± 0.25 ^a^	15.22 ± 1.49 ^a^	290.23 ± 32.06 ^a^
PEF 6 (0.9, 150)	0.90	153.09 ± 0.77	0.53 ± 0.19 ^ab^	14.00 ± 0.60 ^a^	252.93 ± 50.83 ^bc^
One-way ANOVA result			F(8,39) = 4.91	F(8,39) = 108.47	F(6,98) = 9.42
			*p* = 0.00	*p* = 0.00	*p* = 0.00

PEF, pulsed electric fields. * Initial conductivity: 1.61 ± 0.25 mS/cm. ** Initial temperature: 7.22 ± 1.46 °C. All the PEF treatments were carried out at a 20 µs pulse width at 100 Hz. Results are expressed as the mean ± standard deviation of five independent PEF processing experiments. Values in the same column not sharing the same letter are significantly different at *p* < 0.05 analysed with one-way ANOVA and Tukey’s post hoc test.

**Table 2:** Thermal stability and gelatinisation temperature of isolated starch (IS), starch from PEF processing medium (SPM), and potato powder (PP) from PEF treated potatoes.

Thermal Properties	PEF (kV/cm, kJ/kg)							F Value (6, 14)	*p*-Value
	Untreated (No PEF)	PEF 1 (0.5, 50)	PEF 2 (0.7, 50)	PEF 3 (0.9, 50)	PEF 4 (1.1, 50)	PEF 5 (0.7, 150)	PEF 6 (0.9, 150)		
Isolated starch (IS)									
*Tpdw* (°C)	278.35 ± 1.21	278.89 ± 1.60	277.13 ± 1.16	279.06 ± 2.20	277.36 ± 1.13	277.82 ± 1.68	274.25 ± 3.62	0.94	0.50
*To* (°C)	56.67 ± 0.57	56.85 ± 0.79	56.86 ± 0.45	56.52 ± 0.47	56.55 ± 0.37	56.73 ± 0.41	56.67 ± 0.45	0.21	0.97
*Tp* (°C)	59.81 ± 0.50	60.07 ± 0.62	59.94 ± 0.31	59.56 ± 0.33	59.70 ± 0.24	59.82 ± 0.17	59.88 ± 0.23	0.58	0.74
*Tc* (°C)	64.83 ± 1.90	66.13 ± 0.81	65.68 ± 0.31	64.88 ± 0.70	65.21 ± 1.46	65.75 ± 0.61	66.02 ± 0.01	0.80	0.59
*R* (°C)	8.16 ± 1.87	9.28 ± 0.50	8.81 ± 0.45	8.36 ± 0.40	8.67 ± 1.60	9.02 ± 0.88	9.35 ± 0.45	0.55	0.76
*ΔH* (J/g)	33.37 ± 7.09	38.03 ± 1.15	37.74 ± 2.67	39.37 ± 0.50	35.56 ± 6.36	37.69 ± 1.89	39.43 ± 0.21	0.96	0.49
Starch from PEF processing medium (SPM)									
*Tpdw* (°C)	279.78 ± 1.55	280.03 ± 1.35	278.83 ± 1.47	278.02 ± 3.48	276.65 ± 3.00	280.50 ± 0.69	282.30 ± 3.43	1.76	0.18
*To* (°C)	57.69 ± 0.72	57.59 ± 0.54	57.94 ± 0.42	57.97 ± 0.15	58.23 ± 0.13	58.34 ± 0.07	58.23 ± 0.10	1.67	0.20
*Tp* (°C)	60.96 ± 0.67	60.83 ± 0.55	61.09 ± 0.20	60.95 ± 0.19	61.21 ± 0.12	61.20 ± 0.03	61.19 ± 0.03	0.56	0.76
*Tc* (°C)	67.08 ± 0.57	66.56 ± 0.76	66.74 ± 0.25	66.50 ± 0.02	66.57 ± 0.81	66.33 ± 0.07	66.51 ± 0.24	0.72	0.64
*R* (°C)	9.39 ± 0.20 ^a^	8.97 ± 0.23 ^ab^	8.80 ± 0.63 ^ab^	8.53 ± 0.16 ^ab,^*	8.34 ± 0.70 ^ab^	7.98 ± 0.05 ^b,^*	8.27 ± 0.32 ^b,^*	4.33	0.01
*ΔH* (J/g)	37.32 ± 1.97	38.55 ± 1.58	40.08 ± 1.92	40.98 ± 0.51 *	38.89 ± 2.86	38.50 ± 4.09	39.43 ± 0.41	0.84	0.56
Potato powder (PP)									
*Tpdw* (°C)	276.78 ± 3.77	274.40 ± 6.90	273.31 ± 4.63	277.72 ± 1.78	275.21 ± 6.72	276.10 ± 2.69	276.80 ± 4.70	0.31	0.92
*To* (°C)	57.45 ± 0.53	56.99 ± 0.18	56.57 ± 0.57	57.16 ± 0.34	57.42 ± 0.77	56.69 ± 1.06	57.16 ± 0.49	0.88	0.53
*Tp* (°C)	62.08 ± 0.10	61.33 ± 0.10 *	61.10 ± 0.25 *	61.44 ± 0.46	61.61 ± 0.58	61.51 ± 0.64	61.34 ± 0.25 *	1.82	0.17
*Tc* (°C)	67.75 ± 0.42	67.28 ± 0.35	66.64 ± 0.55 *	66.92 ± 0.65	66.86 ± 0.52	66.96 ± 0.91	66.86 ± 0.62	1.13	0.39
*R* (°C)	10.30 ± 0.47	10.29 ± 0.52	10.07 ± 0.87	09.76 ± 0.55	09.44 ± 1.25	10.26 ± 0.76	09.70 ± 0.34	0.65	0.69
*ΔH* (J/g)	26.26 ± 0.23 ^b^	28.21 ± 0.19 ^a,^*	28.31 ± 0.50 ^a,^*	27.41 ± 1.01 ^ab^	20.68 ± 0.87 ^c,^*	28.49 ± 1.09 ^a,^*	26.58 ± 0.25 ^ab^	46.87	0.00

Result expressed as means ± standard deviation of three independent batches of potato sample (10 potatoes per batch). Means in the same row not sharing the alphabets are significantly different at *p* < 0.05 analysed with one-way ANOVA and Tukey’s post hoc test. Means in the same row with asterisk * are significantly different from untreated/No PEF sample (95% interval confidence) analysed with independent *t*-test. *Tpdw*: temperature of peak weight loss, *To*: temperature of onset gelatinisation, *Tp*: temperature of peak gelatinisation, *Tc*: temperature of conclusion gelatinisation, *R*: gelatinisation temperature range, *ΔH*: enthalpy change of gelatinisation.

**Table 3:** Susceptibility, expressed as % (*w/w*) hydrolysed starch, of isolated starch, starch from PEF processing medium, and potato powder from PEF-treated potatoes towards heat-stable α-amylase and amyloglucosidase.

Samples	PEF Treatments (kV/cm, kJ/kg)							F Value (6,14)	*p*-Value
	Untreated (No PEF)	PEF 1 (0.5, 50)	PEF 2 (0.7, 50)	PEF 3 (0.9, 50)	PEF 4 (1.1, 50)	PEF 5 (0.7, 150)	PEF 6 (0.9, 150)		
Isolated starch (IS)	68.62 ± 4.08	65.45 ± 4.39	74.70 ± 3.65	71.23 ± 6.42	67.95 ± 7.91	70.18 ± 6.46	67.86 ± 5.96	0.80	0.58
Starch from PEF processing medium (SPM)	73.09 ± 2.65	78.66 ± 8.74	68.94 ± 3.56	78.78 ± 1.92 *	75.80 ± 3.79	74.53 ± 6.33	83.25 ± 10.9	1.64	0.21
Potato powder (PP)	62.83 ± 6.16	58.73 ± 10.21	61.10 ± 6.55	60.31 ± 5.33	62.28 ± 4.23	58.37 ± 5.71	62.77 ± 8.39	0.22	0.97

Result expressed as mean ± standard deviation (*n* = 3). Values in the same row not sharing the same superscript are significantly different at *p* < 0.05 analysed with one-way ANOVA and Tukey’s post hoc test. Means in the same row with asterisk * are significantly different from untreated/No PEF sample (95% interval confidence) analysed with independent *t*-test.

**Table 4:** Digestibility, expressed as milligram amount of glucose released per mL digest, of isolated starch (IS), starch from PEF processing medium (SPM), and potato powder (PP) from PEF-treated potatoes.

Time (min)	PEF Treatments (kV/cm, kJ/kg)							F Value (6,35)	*p*-Value
	Untreated (No PEF)	PEF 1 (0.5, 50)	PEF 2 (0.7, 50)	PEF 3 (0.9, 50)	PEF 4 (1.1, 50)	PEF 5 (0.7, 150)	PEF 6 (0.9, 150)		
**Isolated starch (IS)**									
0	4.53 ± 0.49	4.75 ± 0.69	4.63 ± 0.25	4.57 ± 0.17	4.55 ± 0.21	4.88 ± 0.50	4.69 ± 0.13	0.61	0.72
20	4.67 ± 0.24	4.81 ± 0.48	4.62 ± 0.26	4.75 ± 0.28	4.67 ± 0.14	5.14 ± 0.41*	4.59 ± 0.21	2.24	0.06
60	4.74 ± 0.14	4.83 ± 0.32	4.86 ± 0.40	4.70 ± 0.14	4.92 ± 0.38	5.06 ± 0.29*	4.92 ± 0.31	1.01	0.43
90	4.68 ± 0.40	4.82 ± 0.48	4.57 ± 0.29	4.52 ± 0.33	4.54 ± 0.25	4.71 ± 0.41	4.60 ± 0.12	0.59	0.74
120	4.46 ± 0.22	4.53 ± 0.30	4.65 ± 0.29	4.49 ± 0.24	4.50 ± 0.21	4.64 ± 0.42	4.46 ± 0.20	0.51	0.80
**Starch from PEF processing medium (SPM)**									
0	4.45 ± 0.23	4.26 ± 0.27	4.36 ± 0.40	4.48 ± 0.19	4.41 ± 0.39	4.09 ± 0.29*	4.24 ± 0.28	1.24	0.31
20	4.47 ± 0.41	4.37 ± 0.23	4.46 ± 0.21	4.43 ± 0.15	4.34 ± 0.33	4.32 ± 0.20	4.33 ± 0.26	0.34	0.91
60	4.61 ± 0.38	4.27 ± 0.14	4.36 ± 0.20	4.45 ± 0.16	4.31 ± 0.26	4.37 ± 0.31	4.43 ± 0.13	1.32	0.28
90	4.55 ± 0.17	4.29 ± 0.30	4.35 ± 0.17	4.33 ± 0.16	4.24 ± 0.15*	4.29 ± 0.06*	4.21 ± 0.25*	2.00	0.09
120	4.35 ± 0.18 ^a^	4.16 ± 0.10 ^ab^	4.17 ± 0.21 ^ab^	4.10 ± 0.23 ^ab^	3.92 ± 0.22 ^b,^*	4.11 ± 0.20 ^ab^	4.06 ± 0.23 ^ab,^*	2.60	0.04
**Potato powder (PP)**									
0	4.71 ± 0.18 ^b^	5.04 ± 0.64 ^ab^	5.13 ± 0.55 ^ab^	5.01 ± 0.30 ^ab^	4.94 ± 0.26 ^ab^	5.02 ± 0.30 ^ab^	5.58 ± 0.39 ^a,^*	2.57	0.04
20	4.71 ± 0.57	4.90 ± 0.43	5.05 ± 0.32	4.95 ± 0.54	5.16 ± 0.28	5.13 ± 0.50	5.38 ± 0.50	1.30	0.28
60	5.25 ± 0.26	4.97 ± 0.39	5.09 ± 0.49	5.31 ± 0.48	5.17 ± 0.19	5.23 ± 0.17	5.41 ± 0.24	1.06	0.40
90	4.88 ± 0.29 ^b^	5.33 ± 0.09 ^a,^*	4.98 ± 0.25 ^ab^	5.13 ± 0.26 ^ab^	5.15 ± 0.27 ^ab^	5.09 ± 0.07 ^ab^	5.30 ± 0.23 ^a,^*	3.02	0.02
120	4.84 ± 0.36	5.18 ± 0.31*	5.01 ± 0.42	5.06 ± 0.15	5.16 ± 0.21	4.96 ± 0.26	5.28 ± 0.30*	1.49	0.21

Result expressed as the mean ± standard deviation of six tests (triplicate samples with duplicate assay). Means in the same row not sharing the same superscript are significantly different at *p* < 0.05 analysed with one-way ANOVA and Tukey’s post hoc test. Means in the same row with asterisk * are significantly different from untreated/No PEF sample (95% interval confidence) analysed with an independent *t*-test.

## Supplementary Materials

The following are available online at https://www.mdpi.com/2304-8158/8/5/159/s1, Video S1: Gelatinisation behaviour of starch from potatoes treated with PEF at an electric field strength of 1.1 kV/cm and energy input of 50.1 kJ/kg (PEF 4) observed under a hot-stage microscopy (at a magnification of 200×) from 30–80 °C at a rate of 5 °C/min.
